# Evaluating the Levels of Mental Stress, Salivary Oxidative Stress, Body Mass Index, and Waist-to-Hip Ratio in University Students With and Without Polycystic Ovary Syndrome and Their Impact on Academic Performance

**DOI:** 10.7759/cureus.71488

**Published:** 2024-10-14

**Authors:** Anjana Goyal, Hemalata Kruthiventi

**Affiliations:** 1 Biochemistry, Manav Rachna Dental College, Manav Rachna International Institute of Research and Studies, Faridabad, IND

**Keywords:** academic performance, academic stress, body mass index, malondialdehyde, mental stress, polycystic ovarian syndrome

## Abstract

Introduction: In recent decades, there has been a significant increase in the number of diseases, with endocrine diseases, such as polycystic ovarian syndrome (PCOS), showing a notable rise. Women with PCOS have a higher risk of depression and stress which can affect the academic performance of the students. Hence, this study aims to compare salivary oxidative stress biomarkers, mental stress levels, body mass index (BMI), and waist-hip ratio (WHR) in students with and without PCOS, and examine the impact of these factors on academic performance.

Methodology: Female students (n=71) with self-reported PCOS (n=36) were compared to those without PCOS (n=35). Perceived and academic stress were assessed using validated scales, while BMI and WHR were measured through physical examination. Academic performance was self-reported and validated by follow-up interviews. Salivary MDA, a biomarker for oxidative stress, was measured using an enzyme-linked immunoassay (ELISA) test.

Result: The analysis revealed that female students with PCOS exhibited higher MDA (ng/ml) levels (0.71±0.13 vs. 0.41±0.14), perceived stress (28.42±4.75 vs. 22.0±6.62), BMI (26.74±6.28 vs. 23.87±4.81), and academic stress (91.03±31.52 vs. 66.86±30.98) compared to those without PCOS. In participants with PCOS, BMI showed a weak positive correlation with academic stress (r=0.312), while the Perceived Stress Score (PSS) and MDA levels showed a strong positive correlation with academic stress (r=0.709 and 0.850, respectively). Academic performance was weakly positively correlated with perceived stress score (r=0.384).

Conclusion: When individual factors (BMI, perceived stress, MDA levels) were compared, positive significance was observed in both PCOS and non-PCOS groups. Findings suggested that female students diagnosed with PCOS experience elevated levels of perceived stress, salivary MDA, and BMI, potentially impacting their academic performance.

## Introduction

Over the past few decades, there has been a drastic increase in the number of diseases in the human race. One of the major causes for this steep increase in the emergence and rise of new disorders is lifestyle changes. The most significant rise is seen in endocrinal diseases. Polycystic ovarian syndrome (PCOS) is seen as the most common endocrinal disease with anxiety symptoms in premenopausal women, showing a 5-10% prevalence rate [1]. One of the most common features of PCOS is considered to be stress [2].

PCOS was formerly known as Stein-Leventhal syndrome, named after the first researcher who identified this disorder. Though the exact etiologic of PCOS is yet to be known, PCOS has a wide spectrum of manifestations such as irregular menstruation, hyperandrogenism, both biochemical and clinical, the appearance of 12 or more cysts/follicles within the range of 2-9 mm in ultrasound, insulin resistance, hypertension, dyslipidaemia and more which all cumulatively can lead to long term complications like type 2 diabetes mellitus, heart disease, infertility. In the past decade, there have been numerous symptoms that have been observed in this syndrome, including acne, alopecia, skin tags, truncal fat, etc. [3- 5].

The cardinal signs of PCOS diagnosis involve hyperandrogenisity- both clinical and biochemical. It is the elevated levels of testosterone in the body that cause the clinical manifestations, such as hirsutism (excessive hair growth), irregular menstruation, or absence of menstruation, and the presence of multiple cysts on ovaries observable under ultrasound sonography (USG) [5].

One of the most common features of PCOS is considered to be stress. This stress can be emotional, oxidative, inflammatory, and metabolic [2]. Studies suggest that mental health and behaviour can affect biological processes and contribute to developing diseases or health risks. Women with PCOS have a higher risk of depression and perceived stress, even after eliminating other factors [6-8]. It is observed in a meta-analysis study that women with PCOS are four times more likely to have depression than those without the condition [4].

In one of the reports, it is stated that obesity plays a pathological role in the development of PCOS [1]. Numerous studies have been done to establish a relationship between PCOS and obesity. It is reported that nearly 38-88% of women suffering from PCOS are either overweight or obese [9,10]. The Northern Finland Birth Cohort (NFBC) has stated that there is an association between body mass index (BMI) and PCOS of all ages. Reports say high adiposity in early childhood is susceptible to developing obesity and PCOS in adulthood [11, 12]. The waist-hip ratio (WHR) and BMI are used to determine central obesity and excess weight. WHO considers WHR above 0.85 in females and BMI above 30.0 as central obesity. Women with PCOS often are overweight or obese, which also contributes to the development of PCOS [13, 14].

Apart from mental stress, it has been observed that oxidative stress is significantly high in women suffering from PCOS. Oxidative stress is a condition in which the antioxidant defense mechanism is vanquished by the number of pro-oxidants accumulated in the body. The serum oxidative stress biomarker malondialdehyde (MDA), is reported to be higher in women with PCOS than those who don't have it [15, 16]. MDA is a compound that is the product of cell membrane lipid peroxidation resulting from the oxidative degradation of polyunsaturated fatty acids due to free radicals. [17].

Numerous studies suggest a potential correlation between MDA levels and PCOS; however, these investigations mainly focus on serum levels, which require invasive procedures. Notably, there is a lack of research examining the relationship between salivary MDA levels and PCOS. Additionally, no studies have addressed the impact of PCOS and associated host factors on students' academic performance. Therefore, this study aims to explore the relationship between MDA levels in the body using a non-invasive method. It is designed to compare the host factors of female students with and without PCOS and to assess the impact of these factors on the academic performance of both groups.

## Materials and methods

A comparative study was designed to compare the impact of MDA levels, BMI, WHR, perceived stress, and academic stress in female university students with and without PCOS. The study was conducted in the Manav Rachna Dental College from 20th August to 20th October, in the year 2022. The study was approved by the Institutional Ethics Committee of the institute (Ref. No. MRIIRS/MRDC/FDS/IEC/2022/18).

Sample size

GPower Software v 3.0 (Heinrich-Heine-Universität Düsseldorf, Düsseldorf, Germany) was used to calculate the sample size for the study. The t-test was chosen for analysis. With an alpha of 0.05, an effect size of 0.88 (based on the difference in salivary oxidative stress biomarker MDA) [18], and a power of 95%, the estimated sample size was 70, with 35 students in each group. To avoid dropouts, the study was undertaken among a sample size of 36 students in each group. Hence, a total of 72 students were included in the study. One participant from the non-PCOS group was not included in the study, because she did not meet the inclusion criteria. Thus, the sample size of students with PCOS was 36 and those without PCOS were 35, totalling 71. The sampling method used was convenience sampling.

Criteria for participant selection

The study included female dental and allied health science students who willingly participated and fell within the age range of 18-25 years. Participants with a history of alcohol or tobacco use, systemic diseases, compromised immune systems, pregnancy, or those taking medications for PCOS were excluded from the study.

Study design

Phase 1

A proforma was distributed to the university's female students. The questionnaire was organized on Google Forms. Based on the results, the population was divided into two groups, those with PCOS (36 individuals), and those without PCOS (35 individuals).

Phase 2

Both groups were given two proformas measuring mental and academic stress. A physical examination was done to measure the host facets such as weight and height for BMI, waist, and hip measurement to measure central obesity. Unstimulated saliva was collected in a saliva collecting tube by passive drool technique from each individual to analyse MDA levels.

Data collection

Before the commencement of the study, the participants were informed about the procedures in the study, they were informed about the study purpose and how and where the collected data would be used, and signed informed consent form was taken from the volunteers.

The proforma for identifying the PCOS population was adapted with permission from a previous study by Goyal et al. (2020) and formatted using Google Forms, which was then circulated among female university students [19].

To measure mental stress, the Perceived Stress Scale (PSS) was used. This is a validated test containing 10 questions. Using a five-point Likert scale, each question was rated from 0-4. ‘0’ is for ‘Never’, ‘1’ is for ‘Almost Never’, ‘2’ is for ‘Sometimes’, ‘3’ is for ‘Fairly Often’, and ‘4’ is for ‘Often’. All the scores were added for the end score which was then analysed.

Academic stress was assessed by the Academic Stress Scale. This scale was taken from a study by Phillips et al. (2020) [20]; it consists of 40 questions and each question has five alternative responses, which are, ‘No Stress (NS)’, ‘Slightly Stress (SS)’, ‘Moderate Stress (MS)’, ‘Highly Stress (HS)’, and ‘Extremely High (EH)’.

The academic performance was assessed by verbally asking and noting the performance of the student in their last academic session in the form of GPA/SGPA (grade point average/semester grade point average).

Saliva Collection

A total of 5 mL of unstimulated saliva was collected in the morning in a sterile universal container. The sample was centrifuged at 3,500 rpm for 10 minutes, and 1.5 mL of supernatant was transferred to Eppendorf tubes, which were kept on ice before being stored at -20°C until further analysis of MDA levels using an enzyme-linked immunoassay (ELISA) test.

Data analysis

According to the responses submitted by the participants, the females suffering with and without PCOS were determined. For perceived stress, the individual scores were assessed. The end score was the deciding factor. The score ranged from 0-40, the higher the score, the higher the perceived stress. The scores were divided into three ranges, 0-13: low stress, 14-26: moderate stress, and 27-40: high stress.

For academic stress, the total number of questions administered was 40, having the possible responses as ‘No Stress (NS)’, ‘Slightly Stress (SS)’, ‘Moderate Stress (MS)’, ‘Highly Stress (HS)’, and ‘Extremely High (EH). Every response was given a score of 0-4. The 40 questions were further sub-grouped into five factors with eight questions each: personal inadequacy, fear of failure, interpersonal difficulties with teachers, teacher-pupil relationship/teaching methods, and inadequate study facilities. The maximum possible score achievable on the Academic Stress Scale is 160, and the highest score for each individual factor is 32. In each factor, the score was added and assessed. The highest score for each factor was 32, i.e. 4 X 8 and 0 was the lowest stress score. The median/moderate stress was 16 (32/2). The higher the calculated score, the greater the academic stress experienced by a student.

The following parameters were measured according to the study design: height and weight for BMI, waist and hip measurements for WHR, and levels of MDA through ELISA. BMI was calculated through weight and height measurement with the formula: 

$BMI = \frac{\text{weight (kg)}}{\text{height}^2 \text{ (m}^2)}$

Central obesity was calculated through the waist and hip ratio.

The level of MDA was measured using a commercially available ELISA kit (MDA ELISA Kit, Elabscience Biotechnology Inc., Houston, TX) according to the manufacturer’s instructions.

Biochemical Armamentarium

The tools used for the salivary MDA test included sterilized containers, test tubes, deionized/distilled water, vortex shaker, incubator, ELISA reader, gloves, micropipette, disposable pipette tips, and absorbent paper.

Statistical analysis

All the data collected was entered into an Excel spreadsheet (Microsoft Corp., Redmond, WA) for further analysis. Statistical analysis was done using SPSS v. 21 (IBM Corp., Armonk, NY). The analysis employed independent t-tests to examine the relationship between PCOS and host factors, while the correlation coefficient was utilized to ascertain their correlation. The data was analysed by taking the alpha as 0.05, the effect size as 0.8, and the power as 95%.

## Results

In this study, participants (N=71) were categorized into two groups: the control group (n=35) and the study group with PCOS (n=36) based on the presence or absence of PCOS. The self-reported responses of participants regarding academic performance, and medical and family history were duly noted in a case study form.

Table 1 shows the demographic details of the participants. Most of the participants were undergraduate students (92.95%), while the postgraduate students constituted 7.04%. The table shows the distribution of participants by age group. The largest group was aged 18-19 years (49.29%), and the smallest was 24-25 years (2.81%). All the participants were female and unmarried.

**Table 1:** Demographic details UG=undergraduate students, PG=postgraduate students

Personal Data		Number	Percentage
Educational Level	U G Students	66	92.95%
	P G Students	05	7.04%
Age (years)	18-19	35	49.29%
	20-21	28	39.43%
	22-23	6	8.45%
	24-25	2	2.81%

Table 2 shows that the participants with PCOS had mean MDA levels of 0.71±0.13 and participants without PCOS had much lower mean MDA levels (0.41±0.14) with a statistically significant difference between the two groups.

**Table 2:** Association of PCOS with MDA The independent t-test was applied; a p-value <0.05 was considered significant. PCOS=polycystic ovarian syndrome; MDA=malondialdehyde; SD=standard deviation

MDA levels (ng/mL)	Participants with PCOS (n=36)	Participants without PCOS (n=35)	p-value
Mean ±SD	0.71±0.13	0.41±0.14	0.0001
Range	0.53-1.03	0.068-0.70	

Participants with PCOS had a higher BMI (26.74±6.28) as compared to participants without PCOS (23.87±4.81) and the difference was statistically significant (p=0.035). Participants with PCOS had higher WHR (0.83±0.07) as compared to participants without PCOS (0.80±0.07) but the difference between the two groups was not statistically significant (p=0.111) (Table 3).

**Table 3:** Association of PCOS with BMI and WHR PCOS=polycystic ovarian syndrome; PSS=Perceived Stress Scale; BMI=body-mass index; WHR=waist-hip ratio;  r=Pearson’s correlation coefficient; SD=standard deviation The independent t-test was applied. A p-value <0.05 was considered significant.

Variable		Participants with PCOS (n=36)	Participants without PCOS (n=35)	p-value
BMI	Mean ± SD	26.74±6.28	23.87±4.81	0.035
	Range	17.88-40.42	15.18-36.77	
WHR	Mean ± SD	0.83±0.07	0.80±0.07	0.111
	Range	0.65-0.96	0.69-0.97	

Participants with PCOS had a higher PSS (28.42±4.75) as compared to participants without PCOS (22.0±6.62) and the difference was statistically significant. The academic stress levels of participants with PCOS were higher (91.03±31.52) as compared to participants without PCOS (66.86±30.98) with a statistically significant difference between the two groups (p=0.002). However, the academic performance of participants with PCOS was less as compared to those without PCOS and the difference was statistically significant (p=0.037) (Table 4).

**Table 4:** Association of PCOS with perceived stress, academic stress, and academic performance PCOS=polycystic ovarian syndrome; PSS=Perceived Stress Scale; SD=standard deviation. The independent t-test was applied. A p-value <0.05 was considered significant.

Variable		Participants with PCOS (n=36)	Participants without PCOS (n=35)	p-value
PSS	Mean ± SD	28.42±4.75	22.0±6.62	0.0001
	Range	16-40	0-34	
Academic stress	Mean ± SD	91.03±31.52	66.86±30.98	0.002
	Range	24-149	8-132	
Academic performance	Mean ± SD	8.17±1.16	8.70±0.95	0.037
	Range	6.02-10.0	7.02-10.0	

As seen in Table 5, when the academic stress and performance were correlated with other independent variables in both participants with PCOS and without PCOS, participants with PCOS, BMI, perceived stress, and MDA levels were found to show a positive significant association with academic stress and academic performance. BMI was found to have a weak positive correlation with academic stress in participants with PCOS (r=0.312). At the same time, PSS and MDA levels showed a strong positive correlation with academic stress (r=0.709 and 0.850 respectively). Academic performance was found to have a weak positive correlation with the PSS (r=0.384).

**Table 5:** Correlation between academic stress and performance with host parameters PCOS=polycystic ovarian syndrome; PSS=Perceived Stress Scale; MDA=malondialdehyde; BMI=body-mass index; WHR=waist-hip ratio;  r=Pearson’s correlation coefficient. A p-value <0.05 was considered significant.

Variable		Academic stress		Academic performance	
		r	p-value	r	p-value
PSS	With PCOS	0.709	0.015	0.384	0.008
	Without PCOS	0.320	0.057	-0.062	0.720
MDA	With PCOS	0.850	0.002	0.584	0.023
	Without PCOS	-0.140	0.416	-0.083	0.629
BMI	With PCOS	0.312	0.015	0.066	0.700
	Without PCOS	0.146	0.402	0.146	0.404
WHR	With PCOS	-0.110	0.522	0.188	0.273
	Without PCOS	0.297	0.083	-0.234	0.175

## Discussion

PCOS is a metabolic disease that can lead to many clinical manifestations over time, e.g. irregular menstruation, hirsutism, and insulin resistance [3, 5].

The study evaluated the levels of mental stress in students with and without PCOS. For the evaluation, the PSS was used. The study demonstrates that the group of students with PCOS experienced significantly higher levels of perceived stress compared to the control group without PCOS. These findings are similar to the study conducted in 2020 by Mondal et al [21]. On the contrary, studies by Khafagy et al. [7], and Zachurzok et al. [22], showed the opposite result wherein the mean value was seen to be higher in those without PCOS than in those with PCOS. This difference could be the results obtained for the different age groups assessed in the studies. While the above-referred studies were mostly concentrating on the adolescent age group, our study was focused on university students.

Although we did not find a significant difference in WHR, it was observed that BMI had a significant difference. These findings follow the previous studies done by Bhattacharya et al. [23], Joshi et al. [24], and Neubronner et al. [25]. Though not significant, our study found the mean value of WHR to be higher in those with PCOS (0.83±0.07) than in those without it (0.80±0.07). A study conducted by Durmus et al. in 2017 measured visceral adiposity and found results similar to those of the current study. They found women with PCOS had higher levels of WHR than those without the condition [26].

Women with PCOS often experience psychological distress due to body dissatisfaction and impaired health-related quality of life, which may lead to depression and anxiety, imposing a significant healthcare burden. The study conducted by Zachurzok et al. found a negative correlation between BMI, PSS-10, and levels of depression and anxiety. This suggests that the underlying issue may not be actual weight, but rather individuals’ perceptions of their weight and its associated implications on mental health [22].

In the present study, MDA levels were much higher in individuals with PCOS (0.71±0.13) than in those without PCOS (0.41±0.14) and the data was highly significant (p=0.0001). A study conducted by Yavuz et al. measured salivary MDA levels using a method involving thiobarbituric acid (TA). Despite the differences in analytical techniques, their findings corroborated the current study results, indicating that MDA levels are elevated in women with PCOS [18]. Elevated serum MDA levels were also found in a similar study, suggesting that oxidative stress may contribute to the metabolic effects of PCOS [27]. According to Smriti et al. [28], salivary MDA levels strongly correlate with serum MDA levels, indicating that systemic oxidative stress affects MDA levels in saliva. The findings of the current study suggest that individuals with PCOS may have high levels of oxidative stress, as evidenced by increased levels of MDA in saliva. MDA is a by-product of lipid peroxidation and can react with proteins, leading to protein malfunction. The accumulation of other reactive carbonyl compounds along with MDA can cause diseases related to oxidative stress, such as diabetes and atherosclerosis [29]. This can be supported by the study by Elting et al. [30], where they showed that women with PCOS had a four times higher prevalence of diabetes. Through this, it can be said that increased MDA levels can predict the risk of developing diabetes in those with PCOS.

Through the responses, it was deduced that those with PCOS had much higher academic stress than those who did not have PCOS. This could be because the group with PCOS had a much higher PSS score than the control group. Through these findings, it was established that the students with PCOS were affected due to their condition.

The positive relation found between PSS and the Academic Stress Scale in students with PCOS suggested that those with higher perceived stress tend to have had high academic stress, which in turn affected their academic performance. This study established that BMI was correlated to academic stress in the participants with PCOS (r=0.312, p=0.015), but was found to be non-significant in those without PCOS. The findings of this study suggested that those with PCOS have had higher MDA levels and that it was correlated to academic stress and academic performance of an individual.

To understand how host factors affect academic performance in students, integrating routine mental health screenings into standard care for those with PCOS is essential, as targeted psychological interventions have been shown to improve mental health outcomes. Incorporating established guidelines would provide a framework for holistic management that addresses both the physical and psychological aspects of the condition. Continued research is also necessary to deepen the understanding of the psychological dimensions of PCOS and to inform effective management strategies.

Limitations

The limitations of the study include its reliance on self-reported information regarding PCOS status, the presence of any other disease/condition, and academic performance. Due to the nature of the population, participants in the study were limited to the age group of 18-26 years, which may limit the applicability of the findings to women of other age groups. Additional research is warranted to further explore these aspects.

## Conclusions

This study compared levels of mental stress, MDA, BMI, and WHR. The impact of these factors was studied in students with and without PCOS to understand the impact on their academic performance. Individuals with PCOS were found to experience higher levels of stress and had elevated MDA in their saliva. A significant difference in BMI was found between individuals with and without PCOS, but there was no significant difference in WHR.

This study's findings suggest that perceived stress, BMI, and MDA are related to academic stress in students with PCOS, and perceived stress and MDA are associated with academic performance. The elevated levels of mental and academic stress are the cardinal points that require a multidisciplinary approach for treating and controlling this syndrome. There is a need for stress management therapies to be introduced in students with PCOS as this condition has a higher chance of affecting their academics and quality of life. 

## Appendices

Appendix 1

Questionnaire to determine the control and study group (Table 6).

**Table 6:** Appendix 1 - PCOS Survey PCOS= Polycystic ovary syndrome

S.No.	Question	Options
1	Email	__
2	I voluntarily consent to participate in this study	Yes
		No
3	Do you have PCOS?	Yes
		No
4	Age:	__ years
5	Weight:	__ kilograms
6	Age of Menarche	
7	In which stream are you?	science (medical/dental)
		Science (non-medical)
8	How many college hours do you have?	less than 8 hours
		more than 8 hours
9	How many hours of sleep do you take?	<8 hours
		8-10 hours
		>10 hours
10	Do you face any trouble falling asleep?	Yes
		No
		On particular days
11	Do you feel stress affects your lifestyle in a major way?	Yes
		No
		Maybe
		Don't know
12	What type of stress do you primarily experience?	Time stress (includes worrying about deadlines or rushing to avoid being late)
		Anticipatory stress (stress experienced when concerned about future)
		Situational stress (in cases of emergencies or certain burdening situations)
		Encounter stress (happens when you worry about interaction with people)
13	Since physical activity is beneficial for health, do you include any physical activity in your daily routine?	Yes
		No
		It depends if I have time
14	Regularity of menstrual cycle	Quite regular
		Irregular, from past few years
		Irregular from the very beginning
15	Length of cycle i.e. for how many days do you bleed?	3 days
		3-5 days
		more than 1 week
16	What type of flow do you have during your periods?	Very heavy
		Heavy
		Light
		Very light (scanty)
17	Do you feel any pain in the lower abdomen i.e. in the pelvic region during your periods?	Yes
		No
		Sometimes
18	When does the pain start?	by the onset of periods
		by the time it ends
		in between
		all the time i.e. from beginning till end
		Only at beginning and end
19	Does it radiate to the posterior part of the lower limbs?	Yes
		No
		Maybe
20	Is the pain severe or bearable?	Severe
		Bearable
21	Did your periods ever occur twice a month?	Yes
		No
		Initially it used to be like this
		Since the past few cycles
22	Do you have a balanced diet?	Yes
		No
		I try to
		Not possible for me, I'm staying somewhere other than home
23	How often do you eat junk food?	Rarely
		A few times
		I can't live without it
24	Are you experiencing any increased or abnormal growth of facial hairs recently?	Yes
		No
25	Do you have a history of acne?	Yes
		No
		Sometimes
26	Are you noticing any kind of stretch marks on your body, especially near the areas of the neck or stomach?	Yes
		No
		Maybe
27	Are you noticing and increased amount of hair fall or thinning of hair recently?	Yes
		No
		Didn’t notice
28	Have you noticed any skin darkening near your neck region?	Yes
		No
29	In the past few days, have you noticed any increase in your anger?	Yes
		No
		A little bit
30	Do you consume tobacco/alcohol in any form?	Yes
		No
31	Do you have any of the following conditions?	Diabetes
		Hypertension
		High cholesterol
		Hypertension
		None
		Other:

Appendix 2: Perceived Stress Scale 

The questions in this scale ask the respondent about their feelings and thoughts during the last month. In each case, they were asked to indicate by circling how often they felt or thought a certain way. The scale has given values, namely: 0 = Never; 1 = Almost Never; 2 = Sometimes; 3 = Fairly Often; 4 = Very Often. The questions are included in Table 7.

**Table 7:** Appendix 2 - Perceived Stress Scale

S.No.	Questions	Values				
1	In the last month, how often have you been upset because of something that happened unexpectedly?	0	1	2	3	4
2	In the last month, how often have you felt that you were unable to control the important things in your life?	0	1	2	3	4
3	In the last month, how often have you felt nervous and “stressed”?	0	1	2	3	4
4	In the last month, how often have you felt confident about your ability to handle your personal problems?	0	1	2	3	4
5	In the last month, how often have you felt that things were going your way?	0	1	2	3	4
6	In the last month, how often have you found that you could not cope with all the things that you had to do?	0	1	2	3	4
7	In the last month, how often have you been able to control irritations in your life?	0	1	2	3	4
8	In the last month, how often have you felt that you were on top of things?	0	1	2	3	4
9	In the last month, how often have you been angered because of things that were outside of your control?	0	1	2	3	4
10	In the last month, how often have you felt difficulties were piling up so high that you could not overcome them?	0	1	2	3	4

Appendix 3: Academic Stress Scale

This scale consists of 40 items describing the stress in the respondents' institution/college life from various sources. The questions are included in Table 8.

**Table 8:** Appendix 3 - Academic Stress Scale

S.No.	Question	Options				
1	Teachers make too many extra demands on students.	No Stress	Slight Stress	Moderate Stress	High Stress	Extreme Stress
2	Poor interest in some subjects.	No Stress	Slight Stress	Moderate Stress	High Stress	Extreme Stress
3	Progress reports to parents	No Stress	Slight Stress	Moderate Stress	High Stress	Extreme Stress
4	The teacher does not humour us.	No Stress	Slight Stress	Moderate Stress	High Stress	Extreme Stress
5	Lack of concentration during study hours.	No Stress	Slight Stress	Moderate Stress	High Stress	Extreme Stress
6	Difficulty in remembering all that is studied.	No Stress	Slight Stress	Moderate Stress	High Stress	Extreme Stress
7	Worrying about the examinations.	No Stress	Slight Stress	Moderate Stress	High Stress	Extreme Stress
8	Lack of self-confidence.	No Stress	Slight Stress	Moderate Stress	High Stress	Extreme Stress
9	The teachers do not listen to our ideas.	No Stress	Slight Stress	Moderate Stress	High Stress	Extreme Stress
10	Conflict with friends/ college authorities.	No Stress	Slight Stress	Moderate Stress	High Stress	Extreme Stress
11	Teachers give more punishment in the class.	No Stress	Slight Stress	Moderate Stress	High Stress	Extreme Stress
12	Worry about results after examinations.	No Stress	Slight Stress	Moderate Stress	High Stress	Extreme Stress
13	Hesitate to ask the teacher for detailed explanation.	No Stress	Slight Stress	Moderate Stress	High Stress	Extreme Stress
14	Biased attitude of the teacher.	No Stress	Slight Stress	Moderate Stress	High Stress	Extreme Stress
15	Inadequate space or room for study at home.	No Stress	Slight Stress	Moderate Stress	High Stress	Extreme Stress
16	Not knowing how to prepare for the examinations.	No Stress	Slight Stress	Moderate Stress	High Stress	Extreme Stress
17	Lack of assertiveness (confidence) in the class.	No Stress	Slight Stress	Moderate Stress	High Stress	Extreme Stress
18	Lack of opportunity to meet teachers.	No Stress	Slight Stress	Moderate Stress	High Stress	Extreme Stress
19	The teacher uses socio-economic status to influence students.	No Stress	Slight Stress	Moderate Stress	High Stress	Extreme Stress
20	Slow in getting along with the curriculum.	No Stress	Slight Stress	Moderate Stress	High Stress	Extreme Stress
21	Exam papers are tough and not valued well.	No Stress	Slight Stress	Moderate Stress	High Stress	Extreme Stress
22	Unable to complete the assignment in time.	No Stress	Slight Stress	Moderate Stress	High Stress	Extreme Stress
23	Lack of communication between teachers and students.	No Stress	Slight Stress	Moderate Stress	High Stress	Extreme Stress
24	Monotonous (boring or tedious) teaching style by the teacher.	No Stress	Slight Stress	Moderate Stress	High Stress	Extreme Stress
25	Not enough discussion in the class.	No Stress	Slight Stress	Moderate Stress	High Stress	Extreme Stress
26	Lack of mutual help among classmates.	No Stress	Slight Stress	Moderate Stress	High Stress	Extreme Stress
27	Lack of fluency while speaking the language other than the mother tongue.	No Stress	Slight Stress	Moderate Stress	High Stress	Extreme Stress
28	Difficulty in public speaking.	No Stress	Slight Stress	Moderate Stress	High Stress	Extreme Stress
29	The teacher is fast and does not use blackboard legibly.	No Stress	Slight Stress	Moderate Stress	High Stress	Extreme Stress
30	Teachers lacking interest in students.	No Stress	Slight Stress	Moderate Stress	High Stress	Extreme Stress
31	Examination syllabus is too heavy in some subjects.	No Stress	Slight Stress	Moderate Stress	High Stress	Extreme Stress
32	Feeling of inferiority.	No Stress	Slight Stress	Moderate Stress	High Stress	Extreme Stress
33	Unable to discuss academic failures with parents.	No Stress	Slight Stress	Moderate Stress	High Stress	Extreme Stress
34	Not able to grasp the subject matter.	No Stress	Slight Stress	Moderate Stress	High Stress	Extreme Stress
35	Incomplete and confusing study material.	No Stress	Slight Stress	Moderate Stress	High Stress	Extreme Stress
36	Eleventh-hour preparation for the examinations.	No Stress	Slight Stress	Moderate Stress	High Stress	Extreme Stress
37	Importance of the subject matter.	No Stress	Slight Stress	Moderate Stress	High Stress	Extreme Stress
38	Difficulty in adjusting with opposite gender.	No Stress	Slight Stress	Moderate Stress	High Stress	Extreme Stress
39	Inadequate subject knowledge of the teacher.	No Stress	Slight Stress	Moderate Stress	High Stress	Extreme Stress
40	Inadequate lab and library facilities.	No Stress	Slight Stress	Moderate Stress	High Stress	Extreme Stress

Appendix 4: patient information sheet

The following table includes questions on on the respondents' medical history and physical characteristics (Table 9).

**Table 9:** Appendix 4 - patient information sheet/case study form PCOS=polycystic ovary syndrome; BMI=body mass index; MDA=malondialdehyde

Variables		
Do you have PCOS?	Yes	No
Do you have any systemic disease?	Yes	No
Do you have any autoimmune disorders?	Yes	No
Are you currently being treated for any sort of illness or medical condition?	Yes	No
Are you pregnant?	Yes	No
PATIENT HISTORY		
Do you have a history of:	Yes	Duration
Smoking	Yes/No	__Years
Alcohol	Yes/No	__Years
Diabetes	Yes/No	__Years
Hyper/hypotension	Yes/No	__Years
Hyper/hypothyroidism	Yes/No	__Years
FAMILY HISTORY		
Do you have a family history of:	Yes	Duration
Diabetes	Yes/No	__Years
Hyper/hypotension	Yes/No	__Years
Heart disease	Yes/No	__Years
Hyper/hypothyroidism	Yes/No	__Years
PCOS	Yes/No	__Years
Perceived Stress Scale:	Score:	
Academic Stress Scale:	Score:	
Academic Performance:	Score:	
BMI		
Weight	___kilograms	
Height	__meters	
Calculated BMI	____kgs/m2	
CENTRAL OBESITY		
Waist length	__inches	
Hip length	__inches	
Waist Hip Ratio	Ratio:	
MDA		
Saliva Sample	MDA Levels:	
